# The Triage and Diagnostic Accuracy of Frontier Large Language Models: Updated Comparison to Physician Performance

**DOI:** 10.2196/67409

**Published:** 2024-12-06

**Authors:** Michael Joseph Sorich, Arduino Aleksander Mangoni, Stephen Bacchi, Bradley Douglas Menz, Ashley Mark Hopkins

**Affiliations:** 1 College of Medicine and Public Health Flinders University Adelaide Australia; 2 Department of Clinical Pharmacology Southern Adelaide Local Health Network Adelaide Australia; 3 Department of Neurology and the Center for Genomic Medicine Massachusetts General Hospital and Harvard Medical School Boston, MA United States

**Keywords:** generative artificial intelligence, large language models, triage, diagnosis, accuracy, physician, ChatGPT, diagnostic, primary care, physicians, prediction, medical care, internet, LLMs, AI

## Introduction

The medical capabilities of large language models (LLMs) are progressing rapidly [1-3]. Benchmarking LLMs against human performance with clinically relevant tasks enables tracking current capabilities and progress. The triage (level/urgency of care to seek) and diagnostic accuracy of the GPT-3 model were recently compared with 5000 lay individuals using the internet and 21 practicing primary care physicians [4]. The triage ability of GPT-3 was significantly inferior to that of physicians, having similar accuracy to lay individuals. The diagnostic ability was close to but below that of physicians [4]. It is uncertain whether more recent frontier LLMs are still inferior to physicians on this benchmark.

## Methods

### Overview

The 48 case vignettes—including both common and severe conditions—validated by Levine and colleagues [4] were evaluated using three LLMs that are typically highly ranked across diverse benchmarks: GPT-4o-2024-05-13 (OpenAI), Claude-3.5-Sonnet (Anthropic), and Gemini-1.5-Pro-001 (Google) via a Python application programming interface. The LLMs were instructed to identify potential diagnoses and provide step-by-step reasoning. Subsequently, they reflected on the reasoning and selected the top three diagnoses in order of likelihood. For triage prediction, the LLM was supplied with the vignette and the three diagnoses it predicted. It was instructed to identify the urgency of the required medical care, including its step-by-step reasoning.

**Figure 1:**
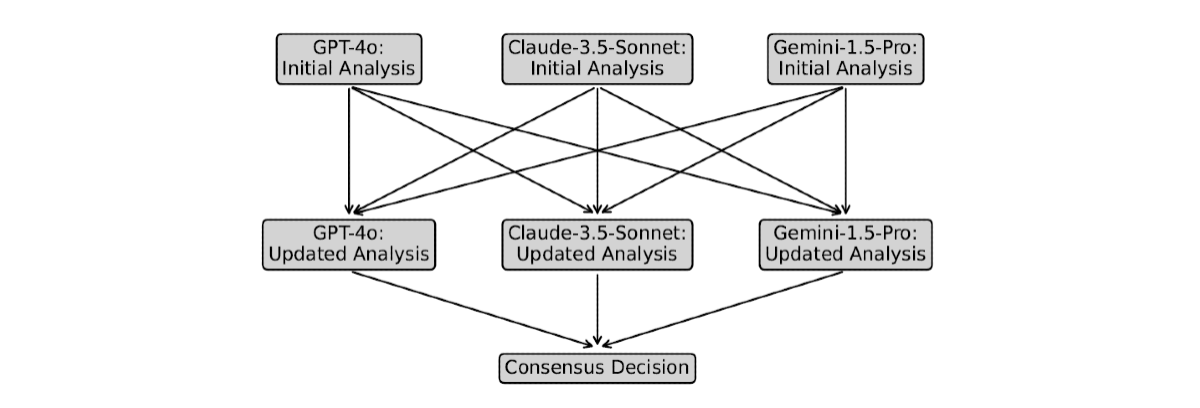
Large language model (LLM) collaboration: a triage/diagnosis workflow involving initial analysis (the LLM’s initial decision and step-by-step reasoning), updated analysis (reflecting on all LLM initial analyses and updating decision if appropriate), and consensus decision (majority vote of the individual LLM’s updated decisions).

A multi-agent workflow involving collaboration between the three distinct LLMs was also evaluated (Figure 1). Each LLM was provided with its initial analysis (decision plus reasoning) and the analyses of the two other LLMs. Each LLM was instructed to reflect on all analyses and update its proposed diagnoses/triage as appropriate. The consensus decision (majority vote) was identified by an independent frontier LLM (Llama-3.1-405B; Meta) to avoid preferencing the output of a specific LLM.

Diagnostic accuracy was evaluated by whether the correct diagnosis was one of the three proposed by the LLM (top 3) [4]. Additionally, the accuracy of the first-ranked diagnosis (top 1) was assessed. Triage was assessed as urgent (emergency department or seeing a doctor within a day) versus nonurgent (seeing a doctor within a week or self-care) [4]. The prompts and LLM settings are provided in Multimedia Appendix 1.

### Ethical Considerations

This study involved a secondary analysis of publicly available synthetic case vignettes. No data on human participants were used. The research was undertaken with approval from the Flinders University Human Research Ethics Committee (project ID 7800).

## Results

The correct diagnosis was among the top three proposed diagnoses for 98.6% (142/144; frontier LLMs) and 100% (48/48; LLM collaboration) of cases. Individually, the performance of GTP-4o, Claude-3.5-Sonnet, and Gemini-1.5-Pro was 98% (47/48), 100% (48/48), and 98% (47/48), respectively.

The most likely diagnosis prediction was correct for 86.8% (125/144; frontier LLMs) and 98% (47/48; LLM collaboration) of cases. Individually, the performance of GTP-4o, Claude-3.5-Sonnet, and Gemini-1.5-Pro was 94% (45/48), 96% (46/48), and 71% (34/48), respectively.

Triage was correct for 92.4% (133/144; frontier LLMs) and 92% (44/48; LLM collaboration) of cases. The most common error was overestimating the urgency. Individually, the performance of GTP-4o, Claude-3.5-Sonnet, and Gemini-1.5-Pro was 92% (44/48), 94% (45/48), and 92% (44/48), respectively.

## Discussion

Contemporary frontier LLMs have substantially improved performance compared to GPT-3 for diagnosis (top three: 142/144, 98.6% vs 42/48, 88%; top one: 125/144, 86.8% vs 31/48, 65%) and triage (133/144, 92.4% vs 34/48, 71%) [4], highlighting the rapid progress in generative artificial intelligence performance. For diagnosis of these clinical vignettes, frontier LLMs performed similarly to physicians (top three: 142/144, 98.6% vs 637/666, 95.6%) [4].

In triaging these clinical vignettes, frontier LLMs (133/144, 92.4%) now perform substantially better than lay individuals (3706/5000, 74.1%) who could use the internet (before the availability of LLMs) and similarly to primary care physicians (608/666, 91.3%) [4]. This capability is consistent with recent evaluations of modern LLMs for emergency department triage [5,6]. A limitation of this study is the relatively small sample size of cases evaluated. Given the encouraging performance of contemporary LLMs for triage assessment, future studies should assess whether LLMs allow lay individuals to make better triage decisions regarding the urgency of care they require.

The rapid progress in LLM capabilities poses challenges for tracking their current capability for health-related tasks. This includes challenges for traditional peer-reviewed publications, which can become outdated by the time of publication.

Additionally, we show that newer techniques involving collaboration between multiple distinct LLMs may improve diagnostic performance. However, this comes at the cost of adding operational complexity. Other methods, such as fine-tuning and in-context learning (eg, integrating search functionality and demonstrations of how to work through complex cases), offer opportunities to improve the performance of LLMs [1,2].

## Appendix

Multimedia Appendix 1 Settings and prompts used for large language models.

## Abbreviations

LLM: large language model

## Data Availability

The case vignettes used are publicly available [4]. The prompts used are available in Multimedia Appendix 1.
