# Supplementary material for: The Triage and Diagnostic Accuracy of Frontier Large Language Models: Updated Comparison to Physician Performance
Source: J Med Internet Res. 2024 Dec 6;26:e67409. doi: 10.2196/67409 (PMC11662182; doi:10.2196/67409)
Supplement: Multimedia Appendix 1 [file jmir_v26i1e67409_app1.docx]

**Supplemental Methods**

**LLM settings:** defaults used for all settings other than temperature set to 0.

**Diagnosis prompt 1:**

You are a senior medical doctor with extensive experience in medical diagnosis. Below is a clinical vignette, and you will need to identify the most likely diagnoses.

# Clinical Vignette:

[INSERT_CURRENT_PROBLEM]

[INSERT_ADDITONAL_DETAILS_LIST]

# Instructions:

Based on the provided clinical vignette, please provide the following information in a manner suitable for a medical audience:

1. List the findings (including both objective and subjective) from the clinical vignette that are relevant for determining the most likely diagnoses.

2. List the most likely diagnoses, up to a maximum of 5 potential diagnoses. For each potential diagnosis, provide the following information in a manner suitable for a medical audience:

- Name the diagnosis using standard medical terminology.

- Provide clear reasoning justifying this potential diagnosis based on the information in the clinical vignette. Also, consider how common the proposed diagnosis is in the general population (relative to other potential diagnoses).

- A proposed causal mechanistic explanation that describes the sequence of cause and effect, starting at the initial underlying cause of the issue and continuing through the presenting signs and symptoms.

- The key pieces of information in the clinical vignette (plus consideration of the general incidence/prevalence of the diagnosis in the population) that help to distinguish the relative likelihood of this diagnosis from others considered.

- The likelihood that this is the correct diagnosis (Highly likely, Likely, Possibly, Unlikely, Very Unlikely).

- List alternative ways of naming or describing this diagnosis. Reflect on the proposed causal mechanism and consider alternative naming of the underlying issue based on the clinical syndrome, pathophysiological outcome or direct etiology.

**Diagnosis prompt 2:**

You are a senior medical doctor with extensive experience in medical diagnosis. Below is a clinical vignette and an initial analysis of potential diagnoses. You will need to critically review the vignette and initial analysis, identify any important errors, and make a final decision on the most likely diagnosis.

# Clinical Vignette:

[INSERT_CURRENT_PROBLEM]

[INSERT_ADDITONAL_DETAILS_LIST]

# Initial Analysis:

[INSERT_INITIAL_ANALYSIS]

# Instructions:

Please provide the following information using language suitable for a medical audience and structured in JSON format:

1. "corrections": An array of any critical errors or omissions in the initial analysis that meaningfully impact the evaluation of the likelihood of each potential diagnosis. Errors or omissions that don't meaningfully impact diagnosis likelihood should be ignored. Each error should be a string justifying the error/omission, the correction, and its importance to the likelihood of diagnosis. If there are no errors, this array should be empty.

2. "duplicate diagnoses": Identify any potential diagnoses that are duplicates of each other in the initial analysis. For example, if two potential diagnoses describe the same underlying issue and causal mechanism, they should be considered duplicates. If there are no duplicates, this array should be empty.

3. "potential diagnoses": An updated array of the three most likely diagnoses, incorporating any identified corrections and duplicate diagnoses. The most likely diagnosis should be listed first. Each potential diagnosis should be an object containing the following:

- "diagnosis" (string): Name the diagnosis using standard medical terminology.

- "justification" (string): Provide clear reasoning justifying this potential diagnosis based on the information in the clinical vignette. Also, consider how common the proposed diagnosis is in the general population (relative to other potential diagnoses).

- "causal mechanism" (string): A proposed causal mechanistic explanation that describes the sequence of cause and effect, starting at the initial underlying cause of the issue and continuing through the presenting signs and symptoms.

- "key differentiators" (string): The key pieces of information in the clinical vignette (plus consideration of the general incidence/prevalence of the diagnosis in the population) that help to distinguish the relative likelihood of this diagnosis from others considered.

- "likelihood" (string): The likelihood that this is the correct diagnosis [Highly likely, Likely, Possibly, Unlikely, Very Unlikely].

   - "alternative naming" (array): List alternative ways of naming or describing this diagnosis. Reflect on the proposed causal mechanism and consider alternative naming of the underlying issue based on the clinical syndrome, pathophysiological outcome or direct etiology.

Please ensure the JSON is properly formatted (e.g. comma separating each attribute-value pair) and uses appropriate data types for each piece of information.

**Diagnosis consensus prompt 1:**

You are a senior medical doctor with extensive experience in medical diagnosis. Below is the clinical vignette, your initial analysis of the most likely diagnoses for the clinical vignette, and the analyses provided independently by two other doctors.

Your task is to critically review your initial analyses and the analyses of the two other doctors and, where appropriate, update your final answer for the most likely diagnoses.

# Clinical Vignette:

[INSERT_CURRENT_PROBLEM]

[INSERT_ADDITONAL_DETAILS_LIST]

# Your initial analysis:

[INSERT_INITIAL_ANALYSIS]

# Doctor 1's analysis:

[INSERT_DOCTOR1_ANALYSIS]

# Doctor 2's analysis:

[INSERT_DOCTOR2_ANALYSIS]

# Instructions:

1. Review and Analysis:

- Critically evaluate the initial analysis and the analyses provided by the two other doctors. Identify which arguments are most convincing for the most likely diagnosis based on the clinical vignette.

- Focus primarily on comparing and evaluating the first-ranked diagnosis in each analysis. Identify concordance or discrepancies among the analyses.

- Provide a comprehensive analysis of the proposed most likely diagnosis in a structured manner, considering all provided analyses.

2. Final Diagnosis and Justification:

- Update the final most likely diagnoses based on your critical review. List the three most likely diagnoses, in order of likelihood, with detailed reasoning.

3. Output Format:

- Provide all outputs in a structured JSON format, including the critical review, and the updated diagnoses with detailed justifications.

- Ensure all sections are included within the JSON output and that it is well-structured and properly formatted.

# JSON Output Structure:

- "analysis" (string): A detailed critical evaluation of the three analyses for the most likely diagnosis. Include arguments supporting or refuting each diagnosis and identify the most convincing diagnosis.

- "potential diagnoses" (array of objects): Your updated list of the three most likely diagnoses, ordered by likelihood. Each object should contain:

- "diagnosis" (string): The diagnosis in standard medical terminology.

- "justification" (string): The step-by-step reasoning that led to this diagnosis being proposed as plausible, including clinical evidence and epidemiological context.

- "inconsistency" (string): Any presenting signs and symptoms that are inconsistent or not well explained by this diagnosis. Present all reasonable arguments against this diagnosis.

- "causal mechanism" (string): Include a clear causal chain from the initial underlying cause to the presenting signs and symptoms. Include all signs and symptoms that can be explained by the proposed causal mechanism. Any signs or symptoms that cannot be explained (or are unlikely to be explained) by the proposed causal pathway for the diagnosis should be explicitly noted at the end.

- "key differentiators" (string): The key pieces of information in the clinical vignette (plus consideration of the general incidence/prevalence of the diagnosis in the population) that help to distinguish the relative likelihood of this diagnosis from others considered.

- "likelihood" (string): The likelihood of this being the correct diagnosis [Highly likely, Likely, Possibly, Unlikely, Very Unlikely].

- "alternative naming" (array): Alternative names or descriptions for this diagnosis.

**Diagnosis consensus prompt 2:**

You are a senior medical doctor with extensive experience in medical diagnosis. Below is the clinical vignette, and analyses of the most likely diagnoses provided by three doctors.

Your task is to consolidate the analyses.

# Clinical Vignette:

[INSERT_CURRENT_PROBLEM]

[INSERT_ADDITONAL_DETAILS_LIST]

# Doctor 1's analysis:

[INSERT_DOCTOR1_ANALYSIS]

# Doctor 2's analysis:

[INSERT_DOCTOR2_ANALYSIS]

# Doctor 3's analysis:

[INSERT_DOCTOR3_ANALYSIS]

# Instructions:

1. Analysis:

- Let's think step by step.

- Critically evaluate the analyses provided by the three doctors.

- Identify whether there is any consensus among the doctors regarding the most likely diagnosis.

- If two or more doctors agree on the most likely diagnosis, this should be considered the final diagnosis.

- If there is no consensus, you should make the final decision. Let's think step by step. Consider the arguments presented by each doctor and assess the strength of the evidence supporting their proposed diagnoses.

- Subsequently consolidate the two next most likely diagnoses.

2. Final Diagnosis and Justification:

- Update the final most likely diagnoses based on your critical review. List the three most likely diagnoses, in order of likelihood, with detailed reasoning.

3. Output Format:

- Provide all outputs in a structured JSON format, including the critical review, and the updated diagnoses with detailed justifications.

- Ensure all sections are included within the JSON output and that it is well-structured and properly formatted.

# JSON Output Structure:

- "analysis" (string): The output addressing all elements specified in the Analysis step of the instruction.

- "potential diagnoses" (array of objects): Your updated list of the three most likely diagnoses, ordered by likelihood. Each object should contain:

- "diagnosis" (string): The diagnosis in standard medical terminology.

- "justification" (string): The step-by-step reasoning that led to this diagnosis being proposed as plausible, including clinical evidence and epidemiological context.

- "inconsistency" (string): Any presenting signs and symptoms that are inconsistent or not well explained by this diagnosis. Present all reasonable arguments against this diagnosis.

- "causal mechanism" (string): Include a clear and detailed causal chain from the initial underlying cause to each of the presenting signs and symptoms. Include all signs and symptoms that can be explained by the proposed causal mechanism and be specific on how each sign/symptom fits into the causal chain. Any signs or symptoms that cannot be explained (or are unlikely to be explained) by the proposed causal pathway for the diagnosis should be explicitly noted.

- "key differentiators" (string): The key pieces of information in the clinical vignette (plus consideration of the general incidence/prevalence of the diagnosis in the population) that help to distinguish the relative likelihood of this diagnosis from others considered.

- "likelihood" (string): The likelihood of this being the correct diagnosis [Highly likely, Likely, Possibly, Unlikely, Very Unlikely].

- "alternative naming" (array): Alternative names or descriptions for this diagnosis.

**Triage prompt:**

You are a senior medical doctor with extensive experience in medical diagnosis and triage. Below are a clinical vignette and an analysis of the most likely diagnoses. You will need to critically review the vignette and likely diagnosis and subsequently identify the most appropriate triage option.

# Clinical Vignette:

[INSERT_CURRENT_PROBLEM]

[INSERT_ADDITONAL_DETAILS_LIST]

# Most likely diagnosis

[INSERT_MOST_LIKELY_DIAGNOSIS]

Justification: [INSERT_JUSTIFICATION_MOST_LIKELY_DIAGNOSIS]

# Alternative diagnoses (less likely, but to be considered):

[INSERT_LESS_LIKELY_DIAGNOSES]

# Instructions:

Please provide the following information structured in JSON format:

1. "Analysis" (string): Let's think step by step. Discuss the urgency of medical care. Primarily utilise the most likely diagnosis provided to guide triage, but also consider the possibility of the alternative diagnoses even though they are less likely. Details in the clinical vignette can be used to provide more nuanced understanding of person's current situation. Identify the key considerations influencing the urgency of seeking medical care. Consider the severity and timeframe of potential consequences without medical attention, the level of medical attention that would be required to prevent these negative consequences, and whether there is a consensus triage reported in the literature for the most likely diagnosis.

2. "Triage" (string): The most appropriate triage option. This should be one of the following options:

- "Let the health issue get better on its own. It most likely doesn't require seeing a doctor"

- "Try to see a doctor within a week. It likely won't get better on its own, but it's also not an emergency"

- "See a doctor within a day. The issue is urgent, but not an emergency"

- "Call 911 or go directly to the emergency room. The issue requires immediate attention"

Please ensure the JSON is properly formatted and uses appropriate data types for each piece of information.
